# Evaluation of a laboratory quality assurance pilot programme for malaria diagnostics in low-transmission areas of Kenya, 2013

**DOI:** 10.1186/s12936-017-1856-2

**Published:** 2017-05-25

**Authors:** Elizabeth Wanja, Rachel Achilla, Peter Obare, Rose Adeny, Caroline Moseti, Victor Otieno, Collins Morang’a, Ephantus Murigi, John Nyamuni, Derek R. Monthei, Bernhards Ogutu, Ann M. Buff

**Affiliations:** 1 Malaria Diagnostics Center, U.S. Army Medical Research Directorate-Kenya, Box 54, Kisumu, 40100 Kenya; 2 Malaria Diagnostics Center, Kenya Medical Research Institute, Box 54, Kisumu, 40100 Kenya; 3 National Malaria Control Programme, Ministry of Health, P.O. Box 19982, Nairobi, 00200 Kenya; 4 Division of Parasitic Diseases and Malaria, Center for Global Health, Centers for Disease Control and Prevention, 1600 Clifton Rd NE, MS A-06, Atlanta, GA 30333 USA; 5 U.S. President’s Malaria Initiative, United Nations Avenue, Village Market, P. O. Box 606, Nairobi, 00621 Kenya

**Keywords:** Malaria, Microscopy, Quality assurance, Accuracy, Laboratory, Kenya

## Abstract

**Background:**

One objective of the Kenya National Malaria Strategy 2009–2017 is scaling access to prompt diagnosis and effective treatment. In 2013, a quality assurance (QA) pilot was implemented to improve accuracy of malaria diagnostics at selected health facilities in low-transmission counties of Kenya. Trends in malaria diagnostic and QA indicator performance during the pilot are described.

**Methods:**

From June to December 2013, 28 QA officers provided on-the-job training and mentoring for malaria microscopy, malaria rapid diagnostic tests and laboratory QA/quality control (QC) practices over four 1-day visits at 83 health facilities. QA officers observed and recorded laboratory conditions and practices and cross-checked blood slides for malaria parasite presence, and a portion of cross-checked slides were confirmed by reference laboratories.

**Results:**

Eighty (96%) facilities completed the pilot. Among 315 personnel at pilot initiation, 13% (n = 40) reported malaria diagnostics training within the previous 12 months. Slide positivity ranged from 3 to 7%. Compared to the reference laboratory, microscopy sensitivity ranged from 53 to 96% and positive predictive value from 39 to 53% for facility staff and from 60 to 96% and 52 to 80%, respectively, for QA officers. Compared to reference, specificity ranged from 88 to 98% and negative predictive value from 98 to 99% for health-facility personnel and from 93 to 99% and 99%, respectively, for QA officers. The kappa value ranged from 0.48–0.66 for facility staff and 0.57–0.84 for QA officers compared to reference. The only significant test performance improvement observed for facility staff was for specificity from 88% (95% CI 85–90%) to 98% (95% CI 97–99%). QA/QC practices, including use of positive-control slides, internal and external slide cross-checking and recording of QA/QC activities, all increased significantly across the pilot (p < 0.001). Reference material availability also increased significantly; availability of six microscopy job aids and seven microscopy standard operating procedures increased by a mean of 32 percentage points (p < 0.001) and 38 percentage points (p < 0.001), respectively.

**Conclusions:**

Significant gains were observed in malaria QA/QC practices over the pilot. However, these advances did not translate into improved accuracy of malaria diagnostic performance perhaps because of the limited duration of the QA pilot implementation.

**Electronic supplementary material:**

The online version of this article (doi:10.1186/s12936-017-1856-2) contains supplementary material, which is available to authorized users.

## Background

In Kenya, malaria accounts for more than 20% of outpatient visits, 19% of hospital admissions, and 3–5% of hospital deaths and is a leading cause of mortality in children less than 5 years of age [1, 2]. Approximately 70% of the population in Kenya lives in areas at risk of malaria transmission [3]. Prompt and accurate diagnosis of malaria is an important component of malaria case management. In 2010, the World Health Organization (WHO) recommended that all patients with suspected uncomplicated malaria should receive a parasitological test prior to treatment [4]. Correct diagnosis of malaria reduces unnecessary treatment with expensive artemisinin-based combination therapy (ACT), helps prevent the development of drug resistance and increases the likelihood of correct treatment for other febrile illnesses [4, 5].

Both the WHO policy on malaria diagnostics and the Kenya National Malaria Strategy 2009–2017 recommend the use of microscopy and quality-controlled malaria rapid diagnostic tests (RDT) for parasitological diagnosis of malaria [3, 4]. Microscopy has been the primary method for malaria diagnosis historically and was available in 56% of health facilities in Kenya in early 2013 [6]. Microscopy requires well-trained microscopists as well as functional equipment, supplies and electricity [5]. Training of staff in centres of excellence can improve the capacity of individual microscopists; however, when trained microscopists return to health-facility laboratories, they often face many challenges such as poor-quality reagents, non-functional equipment, heavy workloads and lack of trust in results by clinicians [5, 7]. These challenges can contribute to the marginal improvements in the performance observed after training. The lack of institutional laboratory quality assurance programmes and structured periodic supportive supervision to identify problems and take corrective actions also contribute to the slow progress toward improving access to quality malaria diagnostics [5, 8].

Malaria RDTs are recommended by WHO due to affordability, availability and accuracy [9, 10]. In early 2013, only 31% of health facilities in Kenya had malaria RDTs, but 76% of health facilities had either functional microscopy or RDTs available [6]. One objective of the Kenya National Malaria Strategy 2009–2017 is scaling and sustaining access to prompt diagnosis and effective treatment to the entire population [3]. Part of the implementation of the national strategy has been to strengthen laboratory diagnosis of malaria across all levels of the health care system and in all epidemiological zones. Beginning in June 2013, the National Malaria Control Programme (NMCP), Ministry of Health (MOH), implemented a pilot malaria diagnostics quality assurance (QA) programme. The QA programme was implemented first in health facilities in low-transmission areas because routine health data showed that over-diagnosis of malaria was common despite a low prevalence of parasitaemia [11, 12]. The trends in improvements and challenges after the pilot phase of the QA programme implementation in low-transmission areas from June to December 2013 are reported.

## Methods

### Study sites

Eighty-three health facilities were purposefully selected for the pilot QA programme which was implemented from June to December 2013 in the low-transmission counties, defined as having an estimated population-adjusted parasitaemia prevalence of <5%, of the Rift Valley (n = 36), Central (n = 33) and Eastern (n = 14) regions of Kenya [1, 13]. The health facilities were widely distributed in 23 (49%) of 47 counties and represented approximately 2% of health facilities in the three low-transmission regions. To be included in the QA programme, the health facility had to provide malaria microscopy services.

### Training

Twenty-eight laboratory personnel were selected by the regional medical laboratory coordinators based on malaria microscopy competence, communication skills and potential to teach and mentor others. These individuals were primarily assigned to primary or secondary public hospital laboratories, held supervisory positions, and attended malaria microscopy refresher training within the last year. To become QA officers, selected laboratory personnel attended a 2-week QA training which consisted of 5 days of malaria diagnostics training and 5 days of formal quality assurance/quality control (QA/QC) methods and laboratory management systems training in accordance with ISO standard 15189. Training was conducted at the Malaria Diagnostics Center (MDC), Kenya Medical Research Institute (KEMRI) and U.S. Army Medical Research Directorate-Kenya, Kisumu, Kenya. The QA officers underwent bi-annual proficiency testing to ensure they maintained the requisite proficiency to cross-check slides and serve as mentors to laboratory personnel in supported health facilities. None of the QA officers required remedial training for poor performance during the implementation period evaluated.

### Key components of QA programme

The MDC provided formal training to the QA officers and NMCP staff in QA/QC methods. The QA officers implemented informal training, mentoring and supportive supervision for health-facility laboratory personnel and simple quality management systems using a standardized checklist in health-facility laboratories to improve malaria diagnosis by both microscopy and RDTs. Table 1 describes the eight key components of the QA programme. The standardized malaria diagnostics QA checklist used by QA officers is Additional file 1.

**Table 1:** Key components of the laboratory quality assurance programme

Component	Example
Personnel training and competencies	Microscopy/malaria RDT trainings, proficiency testing
Essential laboratory equipment and utilities	Functional microscopes, electricity, water
Essential laboratory consumables and supplies	Slides, stain, gloves
Reference materials	National laboratory guidelines, bench and job aides
Internal QA/QC practices	SOP for equipment calibration, slide cross-checking
External QA/QC programmes	Stepwise Laboratory Improvement Process Towards Accreditation (SLIPTA)
Standard operating procedures	Specimen collection, slide preparation
Safety practices	Personal protective equipment use, waste disposal

*RDT* rapid diagnostic test, *QA/QC* quality assurance/quality control, *SOP *standard operating procedure

### QA programme implementation and data collection

Health facilities for the initial pilot were identified by QA officers, in consultation with supervisory laboratory staff, based on the distance from each QA officer’s primary duty location. The QA officers did not administer the pilot QA programme in the facilities to which they were permanently assigned. The three health facilities that were generally closest to the QA officer’s primary duty station and that had malaria diagnostic services were purposefully chosen for QA programme support. Twenty-seven QA officers were assigned three health facilities each for support, and one was assigned two facilities because of distances in a remote area. Therefore, the facilities formed a convenience sample based on travel time for the QA officers. Over the 7-month pilot period in 2013, QA officers made 1-day visits in the months of June, July, November and December to each health facility for a total of four visits per facility.

At the initiation of the programme, each QA officer was given seed commodities (i.e., slides, slide mailers, slide boxes, Giemsa and immersion oil) to distribute to the health facilities during their first QA visit. Each QA officer was expected to cross-check five negative and five weak-positive slides (i.e., ten total slides) for accuracy at each facility during every visit in accordance with national and WHO guidance [5, 14]. Thick films were examined for the presence or absence of parasites; a minimum of 100 high-power magnification fields were examined before the slide was classified as negative [5, 14]. No thin films were collected or examined for parasite density or speciation as part of the QA pilot programme, and no slides were collected during the first visit. Because the QA programme was implemented in low-transmission counties, five weak-positive slides were not always available to cross-check; in such cases, the QA officer collected additional negative slides to ensure 10 slides in total were checked.

Each QA officer completed a standardized checklist with 17 discrete sections that covered the eight QA components during each visit (Additional file 1). The QA officers recorded data on specific indicators and processes via observation and structured questions. The data was used in real time to tailor the interventions the QA officer provided during the visit and was analysed to evaluate the QA pilot. Sections 1–4 covered basic health facility information including staffing, training and infrastructure. Sections 5–7 covered the availability of laboratory equipment, supplies and consumables including RDTs. QA officers observed and visually confirmed the presence or absence and count of laboratory equipment, which they recorded on the checklist. QA officers also visually confirmed the presence of supplies and consumables and asked the supervisory laboratory officer if the laboratory had experienced a stock out of 7 or more consecutive days during the previous 3 months that prevented the laboratory from performing malaria diagnostics. In addition, dates were recorded when available (e.g., last maintenance date for microscopes).

Section 8 of the checklist covered malaria reference materials, which were visually verified as being present or absent and the location of the material was documented. Reference materials included the national malaria policy and guidelines for laboratory, diagnosis and treatment, and quality assurance. Reference materials also verified were jobs aids and standard operating procedures (SOPs). QA officers documented the availability of one SOP for RDT use and nine SOPs related to microscopy: (1) collection of blood samples, (2) preparation of blood films, (3) preparation of buffered water, (4) preparation of Giemsa, (5) preparation of Field stain, (6) staining of blood films, (7) examination of blood films, (8) slide selection for QA/QC, and (9) use, care and maintenance of microscopes. The SOPs for microscopy are described in detail in the national guidelines for parasitological diagnosis of malaria [14]. QA officers documented the availability of one job aid for RDT use and six related to microscopy: (1) malaria microscopy images, (2) sample collection, (3) smear preparation, (4) staining, (5) smear examination and reporting, and (6) slide selection and validation. QA officers additionally documented whether the job aids and SOPs had been updated in the previous 12 months.

Sections 9 and 10 covered internal and external QA practices. Six internal QA processes were evaluated as present or absent by either observation or asking the supervisory laboratory officer structured questions. The six processes were (1) batch testing of stain using positive-control slides, (2) pH meter calibration, (3) slide cross-checking, (4) QA process and results recording, (5) slide filing, and (6) slide storage. For external QA, QA officers asked the supervisory laboratory officer three yes-or-no questions related to external QA programme participation, which were (1) participation in a malaria-specific external QA programme, (2) participation in any external QA programmes [e.g., WHO Stepwise Laboratory Improvement Process Towards Accreditation (SLIPTA) programme], and (3) feedback received from external QA programme. The name or affiliation of the programme conducting the external QA and last validation dates were documented where available. Section 11 covered the laboratory turnaround time for both slide and RDT results; the results are not reported.

Sections 12–14 were observations of laboratory staff preparing patients and slides, staining and reading slides and using RDTs. For slide preparation, there were nine discrete procedures observed and each procedure had between one and seven steps. For slide staining and reading, there were six discrete procedures observed and each had between one and five steps. For RDT use, there were six discrete procedures observed and each had between two and six steps.

The procedures and steps are described in the standardized malaria diagnostics QA checklist (Additional file 1). The QA officers observed the procedures and recorded the number of steps completed correctly by laboratory staff for each. However, the results are not reported because the same laboratory staff were not observed serially over the course of the QA pilot.

Section 15 documented six laboratory safety issues including the presence or absence of two laboratory safety SOPs: infection prevention (i.e., use of personal protective equipment [PPE]) and post-exposure prophylaxis. The QA officers also observed the presence or absence of the following safety practices in the laboratory: (1) availability of material safety data sheets, (2) use of PPE, (3) segregation and disposal of waste, and (4) container labelling. Sections 16 and 17 of the checklist were a summary of findings identified during the QA visit, recommendations and signatures.

Inaccuracies or deficiencies identified by the QA officer were immediately addressed through on-the-job training and mentoring of health-facility personnel during the visit. The findings and recommendations were also shared with the health-facility laboratory manager and head administrator at the completion of each visit. Laboratory managers and administrators were responsible for implementing corrective actions recommended by the QA officer. Completed checklists and cross-checked slides were sent to MDC with a written report that included a summary of findings and recommendations after each visit.

All ten slides cross-checked on the second, third and fourth visits were sent for review by expert microscopists. Because of the large number of slides generated, slides were distributed to three reference laboratories: MDC, National Malaria Reference Laboratory and Kisumu County Vector Borne Disease Laboratory. Reference laboratory microscopists were certified through the WHO External Competency Assessment for Malaria Microscopy scheme. The NMCP and MDC staff conducted one supportive supervision visit with QA officers to 30% of health facilities during the pilot phase.

### Data analysis

Data from all completed checklists was analysed using Stata 12 (StataCorp, College Station, TX, USA). Data collected from the first QA officer visit to each facility in June 2013 was used as the baseline. The final data was collected during the last QA visit in December 2013. Two-by-two tables were developed for the dichotomous variables at baseline and after the intervention. McNemar’s test, a non-parametric test for paired nominal data, was used to determine if the proportions were different before and after the QA pilot programme implementation [15]. P values were calculated for interpretation of statistical significance.

Slide reader data was entered and analysed in Excel 2010 (Microsoft, Seattle, WA, USA) and Graphpad Prism V5.01 (Graphpad Software, CA, USA). The specificity, sensitivity, positive predictive value (PPV), negative predictive value (NPV) and 95% confidence intervals (CI) were calculated; slides read by health-facility laboratory personnel were compared to the reference laboratory and slides read by the QA officers were compared to the reference laboratory. The Kappa statistic (κ) and 95% CIs were also calculated for inter-reader agreement between health-facility laboratory personnel and QA officers compared to the reference laboratory for each visit [16].

### Ethics, consent and permission

This external QA pilot evaluation was conducted from routine monitoring and evaluation data collected as part of the NMCP’s programmatic implementation. Health-facility administrators and laboratory supervisors attended an orientation workshop to familiarize them with the QA programme prior to the start of the pilot. Administrators and laboratory supervisors agreed to participate in the QA programme and provided written acknowledgement of findings and recommendations at each QA visit via the standardized malaria diagnostics QA checklist (Additional file 1). No compensation or incentives were provided to the participating health facilities or any health-facility personnel except the seed stocks of laboratory supplies that were distributed to all participating health facilities as part of the programme. No personal identifying information was collected from patients or laboratory personnel.

## Results

Of the 83 health facilities selected for the pilot phase of the QA programme, 63 (76%) were public, 2 (2%) were private for profit, and 18 (22%) were faith-based. Twenty-three (28%) were dispensaries, 32 (39%) were health centres, 26 (31%) were primary hospitals and two (2%) were referral hospitals. Although the QA programme began with 83 health facilities, by the fourth visit three health facilities had stopped participating. Only data from the 80 health facilities that completed the full pilot period were included in subsequent analysis.

All 80 participating facilities had the capacity to conduct microscopy services. However, not all facilities provided microscopy services during every QA visit due to limitations such as power outages, absent laboratory staff or lack of supplies. During the first QA visit, 52 (65%) performed only malaria microscopy, 6 (8%) offered only malaria RDTs and 22 (28%) performed both microscopy and RDTs. A total of 315 full-time laboratory staff were reported during the first QA visit across 80 facilities; 247 (78%) were laboratory technologists with a minimum of 3 years of formal training (i.e., diploma level) and 68 (22%) were technicians with a minimum of 2 years of formal training (i.e., certificate level). Among the 315 laboratory personnel, 40 (13%) reported malaria diagnostics-related refresher training in the previous 12 months. Nineteen (6%) reported malaria microscopy refresher training, and 21 (7%) reported malaria RDT training.

**Fig. 1:**
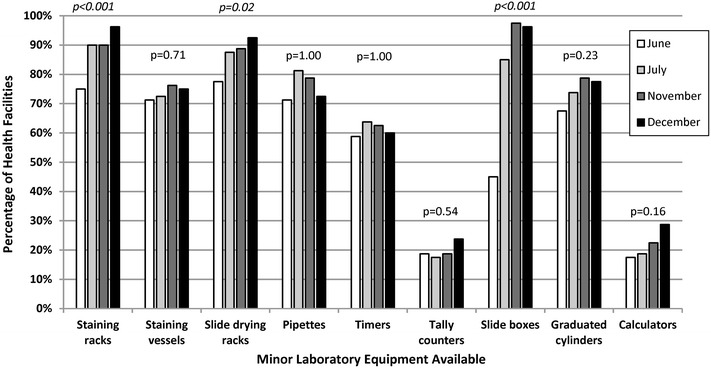
Health facilities with laboratory equipment in malaria low-transmission areas of Kenya, June–December 2013. *Italic* denotes statistically significant improvement from visit 1 to visit 4. Quality assurance officer visually confirmed presence or absence of equipment which was recorded via a standardized checklist

Among the 80 health facilities, the total number of microscopes for all facilities was 194; only 64% (n = 125) of microscopes were functional with an average of 1.8 (range 1–5) functional microscopes per facility. Functional microscopes were defined as having all essential parts and working properly as determined by the QA officers. During the first visit, 70% (n = 56) of facilities documented performing daily equipment maintenance, and by the fourth visit, 85% (n = 68) of facilities documented performing daily equipment maintenance (p = 0.02). Although the majority of health facilities had basic laboratory equipment, less than a third of facilities were observed to have laboratory items such as tally counters and calculators as shown in Fig. 1. The percentage of facilities with staining racks (75–96%, p < 0.001), slide drying racks (78–93%, p = 0.02) and slide boxes (45–96%, p < 0.001) increased between the first and last QA visits.

**Fig. 2:**
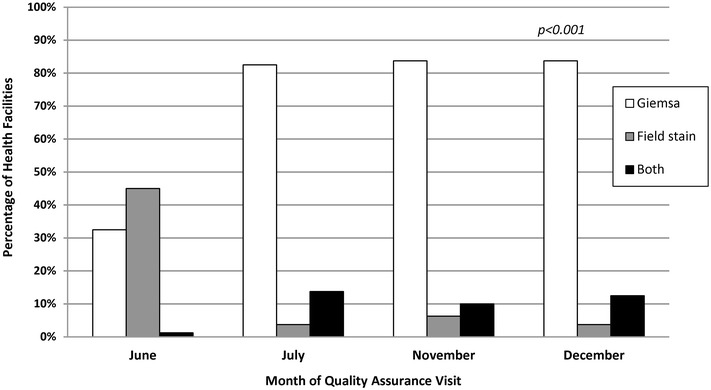
Stain type for malaria microscopy in facilities in low-transmission areas of Kenya, June–December 2013. *Italic* denotes statistically significant improvement from visit 1 to visit 4. Quality assurance officer visually observed type of stain in use which was recorded via a standardized checklist

The availability of general laboratory supplies was relatively high across the four QA visits except for pH paper and RDTs; 43% (n = 34) of facilities had pH paper and 23% (n = 18) had RDTs at the last visit as shown in Table 2. Lancets, methanol, gloves, lens tissue, lens cleaning fluid and Giemsa all significantly increased across the QA pilot. At the first visit, 65% (n = 52) of health facilities had the recommended Giemsa for preparation of blood smears for malaria microscopy. By the fourth visit, 95% (n = 76) of health facilities had Giemsa available (p < 0.001). In addition, health facilities began using Giemsa rather than Field stain for malaria microscopy immediately following introduction by the QA officers as shown in Fig. 2. In June 2013, 33% (n = 26) of facilities were observed using Giemsa, and by December 2013, 84% (n = 67) were using Giemsa (p < 0.001)

**Table 2:** Health facilities with observed laboratory supplies in malaria low-transmission areas in Kenya, June–December 2013

Item	Percentage of health facilities with supply availability, N = 80					
	Visit 1: June	Visit 2: July	Visit 3: November	Visit 4: December	Change in percentage from visits 1 to 4	p value
Lancets	74	84	84	88	14	*0.04*
Methanol	55	79	85	89	34	*<0.001*
Soap	94	96	96	99	5	0.22
Detergent	96	99	100	100	4	0.25
Disinfectant	95	95	99	98	3	0.69
Cotton wool	96	96	96	100	4	0.25
Cotton gauze	91	96	98	96	5	0.34
Pencils	86	93	98	95	9	0.12
Gloves	93	99	100	100	7	*0.03*
pH paper	40	30	40	43	3	0.88
Lens tissue	59	86	93	91	32	*<0.001*
Lens cleaning fluid	46	83	91	90	44	*<0.001*
Slides	94	89	80	94	0	1.00
Immersion oil	99	93	91	100	1	1.00
Giemsa	65	89	86	95	30	*<0.001*
Field stain	89	76	84	81	(−8)	0.29
Rapid diagnostic tests	33	35	29	23	(−10)	0.22

Italic denotes statistically significant improvement from visit 1 to visit 4

(Fig. 2).

**Fig. 3:**
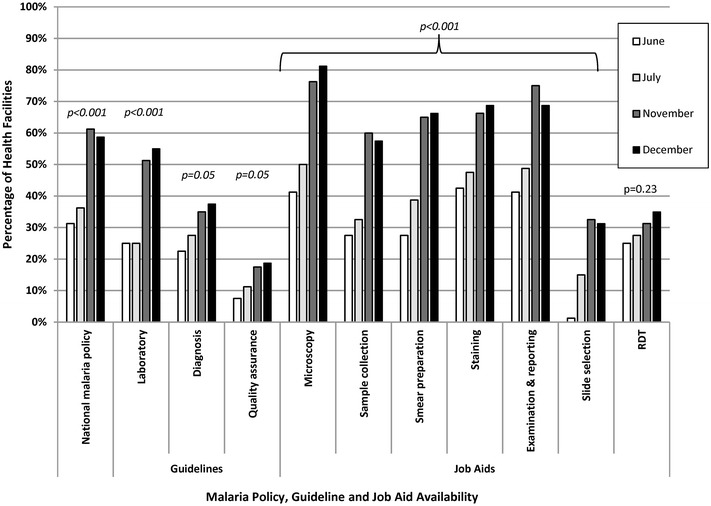
Facilities with malaria reference materials in malaria low-transmission areas of Kenya, June–December 2013. *RDT* rapid diagnostic test. *Italic* denotes statistically significant improvement from visit 1 to visit 4. Quality assurance officer visually confirmed presence or absence of documents and job aids which were recorded via a standardized checklist

Overall, the observed availability of at least one copy each of laboratory reference materials such as the national malaria policy, guidelines and job aids significantly increased from the first to last visits except for the RDT job aid as shown in Fig. 3. Only 31% (n = 25) of health facilities had the national malaria policy at the first visit compared to 59% (n = 47) by the fourth visit (p < 0.001). Only 25% (n = 20) of health facilities had the national malaria laboratory guidelines in June 2013 compared to 55% (n = 44) in December 2013 (p < 0.001). The percentage of facilities with national malaria parasitological diagnosis guidelines and laboratory quality assurance guidelines both significantly increased across the QA pilot (p = 0.05 for both indicators). Across the six malaria microscopy job aids, the mean increase in availability from the first to last QA visit was 32 percentage points (p < 0.001); the largest increase was for the microscopy images job aid (40 percentage points, p < 0.001).

**Fig. 4:**
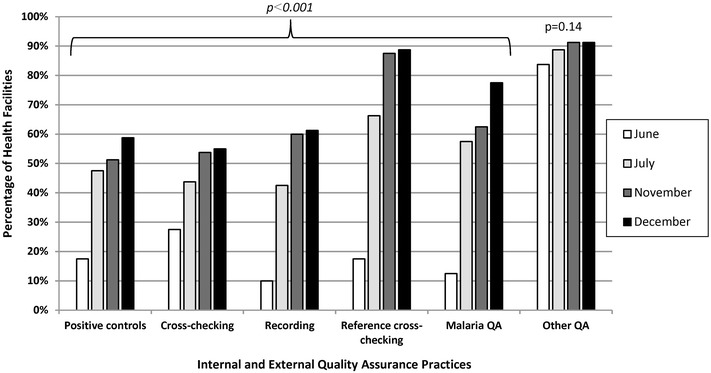
Facilities with laboratory quality assurance practices in malaria low-transmission areas of Kenya, June–December 2013. *QA* quality assurance. *Italic* denotes statistically significant improvement from visit 1 to visit 4. Quality assurance officer visually observed presence or absence of practices and confirmed documentation which were recorded via a standardized checklist

Documentation of internal and external QA activities showed significant increases over the pilot period as shown in Fig. 4. At the first visit, only 18% (n = 14) of facilities were using positive-control slides to check the quality of buffer and stain compared to 59% (n = 47) facilities by the end of the QA pilot (p < 0.001). Initially, 28% (n = 22) of facilities conducted an internal cross-check of slides compared to 55% (n = 44) at the last visit (p < 0.001), and 18% (n = 14) of facilities provided slides for an external reference cross-check compared to 89% (n = 71) at the last visit (p < 0.001). Ten percent (n = 8) of facilities initially were recording internal QA activities compared to 61% (n = 49) at the final visit (p < 0.001). Thirteen percent (n = 10) of facilities reported initially participating in a malaria-specific external QA programme compared to 78% (n = 62) by the final visit (p < 0.001). The percentage of facilities that reported participating in any other laboratory external QA programme did not increase significantly across the QA pilot (p = 0.14).

**Fig. 5:**
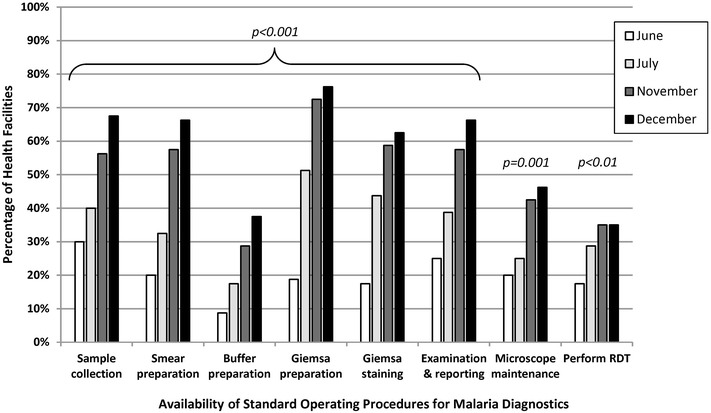
Facilities with malaria diagnostic standard operating procedures in malaria low-transmission areas of Kenya, June–December 2013. *RDT* rapid diagnostic test. *Italic* denotes statistically significant improvement from visit 1 to visit 4. Quality assurance officer visually confirmed presence or absence of documentation which was recorded via a standardized checklist

The percentage of health-facility laboratories with observed SOPs for both microscopy and RDTs also significantly increased across the pilot period as shown in Fig. 5. Across the seven malaria microscopy SOPs, the mean increase in availability from the first to last QA visit was 40 percentage points (p < 0.001); the largest increase was for the Giemsa preparation SOP (57 percentage points, p < 0.001). The percentage of health facilities documented as having the RDT SOP increased from 18% (n = 14) to 35% (n = 28) (p < 0.01). However, despite significant improvements in SOP availability over the QA pilot, less than half of facilities had the buffer preparation (38%), microscope maintenance (46%) and RDT (35%) SOPs by the final visit. Eleven percent (n = 9) of facilities had SOPs that had been updated in the previous 12 months at the start of the QA pilot compared to 38% (n = 30) by the final visit (p < 0.001).

**Fig. 6:**
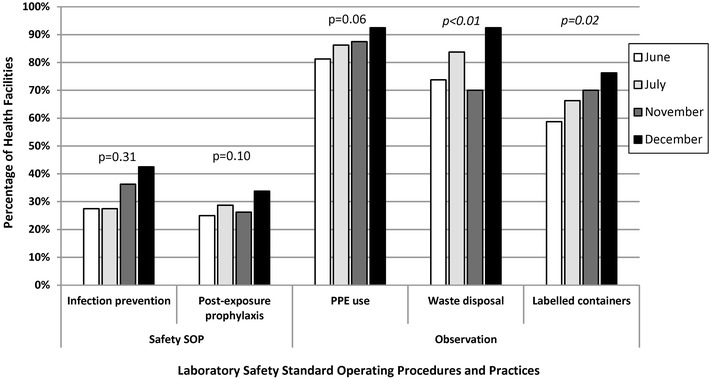
Facilities with laboratory safety procedures and practices in malaria low-transmission areas of Kenya, June–December 2013. *SOP* standard operating procedure, *PPE* personal protective equipment. *Italic* denotes statistically significant improvement from visit 1 to visit 4. Quality assurance officer visually confirmed presence or absence of documentation and observed practices which were recorded via a standardized checklist

As shown in Fig. 6, the availability of two safety SOPs for infection prevention and post-exposure prophylaxis and the observed use of personal protective equipment did not increase significantly over the QA pilot. The QA officers observed a significant increase in proper waste disposal practices (74–93%, p < 0.01) and labelled containers (59–76%, p = 0.02) across the QA pilot.

Malaria blood slides were read by health-facility laboratory personnel, QA officers and reference laboratory personnel, and the results are shown in Table 3. Compared to the reference laboratory, health-facility laboratory personnel read slides with a high specificity (98% at last visit; range 88–98%) and high negative predictive value (NPV) (98% at last visit; range over visits 98–99%). However, health-facility laboratory personnel under-performed in sensitivity (53% at last visit; range over visits 53–96%) and positive predictive value (PPV) (47% at last visit; range over visits 39–53%). The kappa (κ) value, which describes the inter-reader agreement between health-facility personnel and reference laboratory, was 0.48 at the last visit and ranged between 0.48 and 0.66 over the visits. The only statistically significant performance improvement for health-facility personnel over time was for specificity; personnel improved the specificity at which they read slides compared to the reference laboratory by 10 percentage points from 88% (95% CI 85–90%) to 98% (95% CI 97–99%).

**Table 3:** Malaria microscopy diagnostic performance in malaria low-transmission areas in Kenya, June–December 2013

	Health-facility laboratory personnel						Quality assurance officers					
	Visit 2		Visit 3		Visit 4		Visit 2		Visit 3		Visit 4	
Total slides collected	709		408		509		709		408		509	
Slides read by reference laboratory	675	95%	333	82%	509	100%	675	95%	333	82%	509	100%
Reference laboratory	Positive	Negative	Positive	Negative	Positive	Negative	Positive	Negative	Positive	Negative	Positive	Negative
Positive	48	2	8	1	8	7	48	2	8	1	9	6
Negative	75	550	7	317	9	485	44	581	2	322	7	487
Column total	123	552	15	318	17	492	92	583	10	323	16	493

	Health-facility laboratory personnel						Quality assurance officers					
		95% CI		95% CI		95% CI		95% CI		95% CI		95% CI
Sensitivity	96%	86–100%	89%	52–100%	53%	27–79%	96%	86–100%	89%	52–100%	60%	32–84%
Specificity	88%	85–90%	98%	96–99%	*98%*	*97–99%*	93%	91–95%	99%	98–100%	*99%*	*97–99%*
PPV	39%	30–48%	53%	27–79%	47%	23–72%	52%	42–63%	80%	44–97%	56%	30–80%
NPV	99%	99–100%	99%	98–100%	98%	97–99%	99%	99–100%	99%	98–100%	99%	97–100%
Kappa value	0.50	0.41–0.59	0.66	0.43–0.88	0.48	0.27–0.70	0.64	0.55–0.74	0.84	0.66–1.00	0.57	0.36–0.78

*Italic* denotes statistically significant improvement from visit 2 to visit 4

*CI* confidence interval, *PPV* positive predictive value, *NPV* negative predictive value

Compared to the reference laboratory, QA officers also read slides with a high specificity (99% at last visit; range 93–99%) and high NPV (99% across all visits). The QA officers also under-performed compared to the reference laboratory in sensitivity (60% at last visit; range over visits 60–96%) and PPV (56% at last visit; range over visits 52–80%). The ĸ value was 0.57 at the last visit and ranged between 0.57 and 0.84 over all visits. The only statistically significant performance improvement for QA officers over time was also for specificity; QA officers improved the specificity at which they read slides compared to the reference laboratory by six percentage points from 93% (95% CI 91–95%) to 99% (95% CI 97–99%).

## Discussion

Overall participation in the QA pilot implementation was high. Only one QA officer and three health facilities initially selected did not complete the QA pilot. One QA officer, who worked in the most remote geographic location and was assigned two health facilities, opted out of the programme for security and travel-related reasons. One hospital decided not to participate after starting the QA pilot programme because the administration believed that the programme was punitive despite attending the orientation workshop and subsequent briefings and reassurances by laboratory and NMCP supervisors.

Similar to the situation in other sub-Saharan African countries, the evaluation demonstrated a lack of microscopy equipment and trained staff at the health-facility level across a wide geographic area in Kenya [17–19]. Despite national and WHO guidelines that recommend laboratory personnel attend regular malaria diagnostic training to ensure proficiency, fewer than one in seven laboratory staff had completed any malaria diagnostic training and only one in 17 had completed malaria microscopy training in the 12 months before the QA pilot commenced [5, 14]. Although the majority of facilities had more than one functional microscope, over one-third of microscopes across all facilities were not in working condition. The majority of facilities were lacking essential microscopy laboratory equipment such as tally counters and calculators. Overall, almost all health facilities had supplies of general laboratory commodities beyond the small seed stocks from the QA pilot. Historically in Kenya, health facilities charged a small fee for malaria microscopy (i.e., 50 Kenya Shillings or approximately $0.60 in 2013) and other laboratory tests, which was used, in part, to procure laboratory consumables. However, in July 2013, public health-facility fees were removed by government order. Although the removal of health-facility fees seems to have not had a substantial negative effect on the supply of laboratory consumables available at the facility level in the short term, it is unclear if laboratory supply levels will remain adequate and stable in the longer term.

The primary goal of the QA programme was to improve the accuracy of malaria diagnosis by microscopy and malaria RDTs in the supported health facilities. For malaria diagnosis by microscopy, the findings demonstrate no overall performance improvement by health-facility laboratory personnel during the 7-month QA pilot. Based on the observations, routine microscopy in low-transmission settings has high specificity (98%) and NPV (98%), which should provide confidence among clinicians that negative slides are truly negative. More problematic are the sensitivity and PPV of routine microscopy in low-transmission settings. The overall slide positivity of persons presenting with suspected malaria at health facilities ranged from 3 to 7%, which is consistent with other estimates in low-transmission areas of Kenya [12, 19, 20]. The sensitivity was high in the second and third QA visits, 96 and 89%, respectively, but decreased to just 53% during the fourth QA visit in December 2013. Thus, in December 2013, only one of every two slides read as positive was truly positive, leading to likely over-treatment of malaria and under-diagnosis and under-treatment of the actual cause of fever and associated symptoms.

The December 2013 sensitivity might be an outlier and explained by a combination of factors outside the control of the QA programme. In early 2013, Kenya devolved responsibility for health service delivery to the county governments as set forth in the 2010 Constitution of Kenya. By the last quarter of 2013, counties were validating the employment status of health workers, which resulted in many health workers returning to their government-assigned facilities, and counties were beginning to hire additional health workers to fill vacancies. Therefore, factors affecting the diagnostic performance indicators in December 2013 likely included high staff turnover from returning and newly hired laboratory personnel who did not benefit from previous QA visits, a health worker strike from December 2013 to January 2014, and temporary staff hired over the holiday season as described by Wafula et al. in another external QA programme in western Kenya [21].

While the low prevalence of malaria helps to explain the low PPVs (range 39–53%) and high NPVs (98–99%) observed, the kappa value (range 0.48–0.66) for inter-reader agreement between health-facility laboratory personnel and reference laboratory personnel indicates only moderate agreement. The performance of QA officers, who were the “technical experts” providing on-the-job training and mentoring to health-facility laboratory personnel, was only marginally better than health-facility laboratory personnel across all microscopy test performance measures. Therefore, the results of the pilot QA programme support national and WHO recommendations of using malaria RDTs in outpatient facilities (i.e., dispensaries, health centres and hospital outpatient clinics) in low-transmission settings and using malaria microscopy for inpatient case management of complicated cases (i.e., severe malaria or suspected treatment failures) [9, 22]. Evidence from Senegal demonstrates that the use of malaria RDTs is achievable on a national scale with associated improvements in case management outcomes [23].

Over the course of the QA pilot, the performance of RDT indicators lagged compared to microscopy indicators, which was unexpected. The causes might have included lack of RDT availability, limited time for on-the-job training or lack of prioritization of RDT training by QA officers. The QA officers spent 1 day per month at each facility and might have focused more on microscopy and general laboratory QA/QC training since they were selected because of their technical expertise in these areas. Support for the latter is demonstrated by the significant overall improvements in microscopy and QA/QC practice indicators. Due to a fire at the central medical stores in January 2013 that destroyed over 4 million malaria RDTs and other health commodities, only one-third of facilities had malaria RDTs in June 2013 and less than a quarter had RDTs by December 2013. As a result, the majority of facilities did not have RDTs available for patient care or training during the QA pilot.

The QA pilot had a substantial positive impact on the availability of laboratory reference materials and SOPs in health-facility laboratories. Over the course of the 7-month QA pilot, the largest gains were observed in the availability of microscopy job aids and SOPs. The QA pilot also positively impacted internal and external QA practices such as cross-checking slides and recording QA activities across health facilities. Health facility participation in other laboratory external quality assurance programmes did not change significantly over the course of the QA pilot, which suggests that the malaria diagnostics QA pilot was responsible for the improvements observed.

To help meet the overall goal of the QA programme to strengthen malaria diagnostic accuracy, the QA pilot introduced Giemsa in health-facility laboratories that had historically used Field stain. Both the NMCP and WHO recognize Giemsa as the stain of choice for malaria laboratories [5, 14]. Giemsa is the preferred stain for routine malaria microscopy because it can be used to prepare both thick and thin blood films, the stain powder is stable during storage and it has a consistent, reproducible staining quality over a range of temperatures [5]. Giemsa is relatively more expensive than Field stain, which might limit its use by health facilities. To encourage uptake, QA officers were given seed stocks of Giemsa to deliver during the initial QA visit; the Giemsa seed stock was expected to last for several months. Based on the evidence, health facilities had availability and were preferentially using Giemsa for malaria microscopy by the end of the QA pilot implementation period.

The QA pilot had a number of limitations. First, health facilities were not randomly selected for participation in the QA programme thus introducing selection bias. Health facilities were purposefully chosen based on the distance from the duty location of the QA officer in order to decrease travel time and expenses. As a result, the convenience sample of selected health facilities is likely not representative and the findings might not be generalizable to the whole population of health facilities in low-transmission regions of Kenya. Second, the QA pilot was only 7 months in duration and coincided with a tumultuous period in the health system as a result of the devolution of government health services in 2013. A longer pilot and evaluation period would have allowed for more observation points and better estimates of performance outcomes. The last observation point was also in a holiday month when health workers were on strike, and there were substantial staffing changes happening concurrently. Therefore, the observations from December 2013 might have been outliers and not reflected the true impact of the QA programme.

Other limitations were related to the scope, ownership and timing of the QA pilot. The QA officers were laboratory supervisors from primary and secondary hospitals with high workloads and many responsibilities in their assigned positions. The QA officers devoted 1 day per month to each of the supported facilities, which limited the time available for one-on-one training and mentoring, particularly in larger facilities with more laboratory staff. The facility laboratory managers and administrators were responsible for implementing QA officer recommendations, but during the last half of 2013, facility administrators were faced with many other pressing priorities such as validating staff employment, identifying staffing gaps and hiring priorities, eliminating service fees and commodity shortages resulting from devolution of health services.

## Conclusions

The quality of routine malaria microscopy in health facilities in Kenya remains below accepted international standards. The observations from the QA pilot are consistent with findings from another recent external QA programme implemented in a limited number of rural health facilities in western Kenya and provide additional evidence for a coordinated approach to strengthening malaria diagnostics across the larger clinical and laboratory systems [21, 24]. Significant gains were observed in malaria diagnostic QA/QC practices over the pilot. However, these advances did not translate into the primary programme goal of improving accuracy of malaria diagnostic performance perhaps because of the timing and limited duration of the QA pilot implementation.

## Additional file

**Additional file 1.** Malaria diagnostics quality assurance checklist. Standardized checklist used for data collection by quality assurance officers.

## Abbreviations

ACT: artemisinin-based combination therapy
CI: confidence interval
KEMRI: Kenya Medical Research Institute
MDC: Malaria Diagnostics Centre
MOH: Ministry of Health
NMCP: National Malaria Control Programme
NPV: negative predictive value
PPE: personal protective equipment
PPV: positive predictive value
QA: quality assurance
QC: quality control
RDT: rapid diagnostic test
SOP: standard operating procedure
SLIPTA: Stepwise Laboratory Improvement Process Towards Accreditation
WHO: World Health Organization
