# Supplementary material for: Evaluation of a laboratory quality assurance pilot programme for malaria diagnostics in low-transmission areas of Kenya, 2013
Source: Malar J. 2017 May 25;16:221. doi: 10.1186/s12936-017-1856-2 (PMC5445328; doi:10.1186/s12936-017-1856-2)
Supplement: Supplementary file 1 — Additional file 1. Malaria diagnostics quality assurance checklist. Standardized checklist used for data collection by quality assurance officers. [file 12936_2017_1856_MOESM1_ESM.pdf]

MALARIA DIAGNOSTICS QUALITY ASSURANCE CHECKLIST

1. Health Facility / Laboratory Information

**Name of health facility**	
City/Town	
County	
Level of health facility	☐ Level 2 ☐ Level 4 ☐ Level 6<br>☐ Level 3 ☐ Level 5
Ownership	☐ Public ☐ Private ☐ Others<br>☐ Faith Based ☐ Military Specify ☐
Address (P.O. Box)	
Telephone/Fax	
E-mail	
Head of health facility	
Telephone/Fax	
E-mail	
Head of laboratory	
Telephone/Fax	
E-mail	
Hrs of operation HF: weekdays	
Hrs of operation lab: weekdays	
Date of visit (Month/Year)	
Supervision Round	☐ 1 ☐ 2 ☐ 3 ☐ 4

Has this site ever been visited by any laboratory supervisory team in the past 3 months?

☐ Yes
☐ No
☐ Unknown

2. Human Resources - Laboratory Staff

Number of laboratory staff: Note the number of full-time, part-time or casual employees
Tick "N/A" if staff type is not employed at this facility

	Full-time	Part-time	Casual	N/A
Laboratory technologist				
Laboratory technician				
Students on attachment				
Number of staff above that perform malaria diagnostics				
Number of support staff				

3. Refresher Training of Laboratory Staff

Laboratory Staff

Number of **laboratory** staff who attended refresher training in laboratory malaria diagnostics (microscopy, RDT) during the previous calendar year (January - December)

☐ **Number trained**

☐ **No trainings attended (tick)**

1. Name of course attended or certification received ☐ **Date** (Month/Year) ☐ **Duration in hours**

Who provided the training? ☐

2. Name of course attended or certification received ☐ **Date** (Month/Year) ☐ **Duration in hours**

Who provided the training? ☐

3. Name of course attended or certification received ☐ **Date** (Month/Year) ☐ **Duration in hours**

Who provided the training? ☐

4. Water and power supply

Water Supply

Rain water	☐ Yes	☐ No
Well/Borehole	☐ Yes	☐ No
Creek/stream/river	☐ Yes	☐ No
Bottled water	☐ Yes	☐ No
Is there any problem with water supply?	☐ Yes	☐ No
Power supply	☐ Yes	☐ No
Main	☐ Yes	☐ No
Generator	☐ Yes	☐ No
Solar	☐ Yes	☐ No
Fuctional back-up generator	☐ Yes	☐ No
Do power cuts interfere with the ability to perform laboratory malaria diagnosis?	☐ Yes	☐ No
Is the number of power sockets in the lab sufficient?	☐ Yes	☐ No
Is there any problem with power supply?	☐ Yes	☐ No

If yes comment

5. Laboratory Equipment

Number of functional microscopes in use in the health facility

Total number of microscopes (functional AND non-functional)

Is care and maintenance of microscopes routinely done by laboratory staff ?

☐ **Yes** ☐ **No** ☐ **Don't Know**

Is service and repairs of microscopes performed by engineers or contractors? ☐ **Yes** ☐ **No**

If yes, when was the last service or repair performed?

From the availbale records, what is the frequency of service/repairs?

Frequency ☐ **Monthly** ☐ **Quarterly** ☐ **Semi-annually** ☐ **Annually** ☐ **Unknown** ☐ **Does not occur**

Date(Month/Year)

Comment

Weighing scale	Functional	☐	Non-functional	☐
pH meter	Functional	☐	Non-functional	☐

6. Minor Laboratory Equipment

Tick the box that most closely approximates the number of items found in the lab					
Staining racks	☐ 0	☐ 1-2	☐ 3-5	☐ 6-9	☐ 10+
Staining vessels	☐ 0	☐ 1-2	☐ 3-5	☐ 6-9	☐ 10+
Slide drying racks	☐ 0	☐ 1-2	☐ 3-5	☐ 6-9	☐ 10+
Pipettes	☐ 0	☐ 1-2	☐ 3-5	☐ 6-9	☐ 10+
Timers	☐ 0	☐ 1-2	☐ 3-5	☐ 6-9	☐ 10+
Tally counters	☐ 0	☐ 1-2	☐ 3-5	☐ 6-9	☐ 10+
Slides storage boxes	☐ 0	☐ 1-2	☐ 3-5	☐ 6-9	☐ 10+
Graduated cyclinder (50-500mL)	☐ 0	☐ 1-2	☐ 3-5	☐ 6-9	☐ 10+
Calculators	☐ 0	☐ 1-2	☐ 3-5	☐ 6-9	☐ 10+

7. Stock-outs

Did this laboratory experience a stock-out lasting 7 or more consecutive days during the last three months that prevented the laboratory from performing malaria diagnostics?

☐ Yes
☐ No
☐ Unkwnown

If this facility experienced a stock-out, please tick which materials listed below were out of stock			
RDTs	☐ **Out of stock**	Lancets	☐ **Out of stock**
Microscope Slides	☐ **Out of stock**	Methanol	☐ **Out of stock**
Immersion oil	☐ **Out of stock**	Ethanol (96% methylated)	☐ **Out of stock**
Pensils	☐ **Out of stock**	Soap	☐ **Out of stock**
Gloves	☐ **Out of stock**	Detergents	☐ **Out of stock**
pH paper	☐ **Out of stock**	Disinfectants	☐ **Out of stock**
Giemsa stain A	☐ **Out of stock**	Lens tissue	☐ **Out of stock**
Field stain A	☐ **Out of stock**	Lens cleaning fluid	☐ **Out of stock**

Field stain B	Out of stock	Cotton wool	Out of stock
Filter papers	Out of stock	Gauze	Out of stock

Cont' of No.7

What is the primary source of this facility's laboratory supplies and consumables?
1. Hospital procurement
2. Direct purchase from vendors

What procedure does this facility use to order laboratory consumables, reagents and chemicals?
1. Tendering
2. Cash
3. Central pulling system (District/National)
4.Others/Specify

8. Malaria Reference Materials

Only tick "YES" if you can verify that the following materials are physically available

Standard Operating Procedures

Collection of blood sample	Yes	No	If "yes",location
Preparation of thick and thin blood films	Yes	No	If "yes",location
Preparation of buffered water	Yes	No	If "yes",location
Preparation of Giemsa stain	Yes	No	If "yes",location
Preparation of Field stain	Yes	No	If "yes",location
Staining thick/thin blood films with Giems	Yes	No	If "yes",location
Examination and reporting	Yes	No	If "yes",location
Slide selection for QC	Yes	No	If "yes",location
Use, care and maintenance of microscop	Yes	No	If "yes",location
Use of Rapid Diagnostic Tests (RDTs)	Yes	No	If "yes",location

Have the SOPs been updated within the past 12 months? Yes No Unknown

Policies/Guidelines

Malaria Policy
National Guidelines for Laboratory Diagnosis of Malaria
Guidelines for Diagnosis, Treatment and Prevention of Malaria in Kenya
Malaria Quality Assurance Manual
Others/Specify

Bench / Job Aids

Malaria microscopy images	Yes	No	If "yes",location
Sample collection	Yes	No	If "yes",location
Smear preparation	Yes	No	If "yes",location
Staining	Yes	No	If "yes",location
Smear examination and reporting	Yes	No	If "yes",location
Slide selection and validation	Yes	No	If "yes",location
Malaria RDTs	Yes	No	If "yes",location

Have the bench/job aids been updated within past 12 months? Yes No Unknown

9. Internal Quality Assurance

Question			
Are positive control slides available for testing new batches of stain?	☐ yes	☐ No	☐ Unknown
Are standards used to calibrate pH meter?	☐ yes	☐ No	☐ Unknown
Are stained slides rechecked by a person in the laboratory?	☐ yes	☐ No	☐ Unknown
Are results from QA exercises recorded in a register?	☐ yes	☐ No	☐ Unknown
Are slides stored for rereading?	☐ yes	☐ No	☐ Unknown
Are slides stored in slide boxes?	☐ yes	☐ No	☐ Unknown

10. External Quality Assurance

Does the laboratory participate in a malaria EQA scheme?

☐ **Yes** ☐ **No** **Name/affiliation of monitors** ____________ **Date of last validation** ______
(Month/Year)

Does the laboratory participate in any other EQA schemes (HIV/TB or others)?

☐ **Yes** ☐ **No** **Date of last validation** ______
(Month/Year)

Name/affiliation of monitors ____________

Does the laboratory receive feedback for its EQA schemes? ☐ **Yes** ☐ **No** ☐ **Unknown**

11. Turnaround Time for Malaria Test Results

	**Test type**		**Turnaround time in minutes**
**1.**	☐ **RDT**	☐ **Blood Slide**	
**2.**	☐ **RDT**	☐ **Blood Slide**	
**3.**	☐ **RDT**	☐ **Blood Slide**	

Average turnaround time in minutes: RDT ______

Average turnaround time in minutes: BS ______

12. Observation: Preparation of thick and thin blood smears

Observation 1

Procedure

	Steps Correct
1. Patient preparation [**6**]	
2. Specimen collection (finger stick) [**5**]	
3. Specimen collection (venipuncture) [**7**]	
4. Spreading thin films [**6**]	
5. Spreading thick films [**5**]	
6. Labeling [**1**]	
7. Disposal of infectious material [**2**]	
8. Slide drying [**1**]	
9. Slide storage [1]	

Comments

Indicate the stain used

☐ **Giemsa**

☐ **Field**

☐ **Both**

13. Observation: Staining and Reading of Thick and Thin Blood Films

Observation 1

Procedure

Procedure	Tasks Correct
1. Preparation of of Giemsa working solution [3]	
2a. Giemsa staining procedure[**4**]	
2b. Field staining procedure [5]	
3. Slide drying [**1**]	
4. Slide examination [5]	
5. Result reporting [**4**]	
6. Slide cleaning, slide & microscope storage [**4**]	

Comments

Indicate the stain used

- ☐ **Giemsa**
- ☐ **Field**
- ☐ **Both**

14. Observation: RDT

Observation 1

**Procedure**	Tasks Correct
1. RDT preparation [**6**]	
2. Patient preparation [**5**]	
3. Blood collection + dispensing [**5**]	
4. RDT procedure + reading results [**5**]	
5. Recording results [**2**]	
6. Disposal of infectious material [**2**]	

Comments

15. Laboratory Safety Practices

1. Is there an SOP for PEP?	Yes	☐	No	☐
2. Is there an SOP for infection prevention?	Yes	☐	No	☐
3. Are there MSDSs in the laboratory?	Yes	☐	No	☐
4. Are PPE worn during laboratory procedures?	Yes	☐	No	☐
5. Are wastes segregated and disposed appropriately?	Yes	☐	No	☐
6. Are waste disposal containers correctly labeled?	Yes	☐	No	☐

16. Issues Identified and Recommendations

Guidance for the Outreach Training and Support Supervision Report

Please attach a copy of your summary report to the completed OJT checklist(s) and present to NPHLS/NMRL/DOMC
No more than 1-2 pages - preferably typed, otherwise hand written in pen

I. General Information
   - Include the name(s) of the Supervisor(s) conducting the site visit
   - Include the names of the facilities being visited
   - Include the dates of the visits

II. Narrative
   a. describe specific corrective action taken during your visit;
   b. present overall comments and impressions on the state of the facility, staffing, training, quality assurance, and documentation
   c. present challenges faced by the laboratory staff and briefly report on their impressions

of the OTSS process

d. propose recommendations for improvement

17. Signatures

Head of health facility	Supervisor
	Supervisor's place of employment
Head of laboratory	Tel E-mail
Signature Date	Signature Date

Malaria Slide Validation _For QA Officers and Reference Laboratory Centers

	Lab Results	Ref Lab Results	Agreement / Disagreement	Parasites/uL Lab	Parasites/uL Ref Lab
1.	Pos Neg	Pos Neg	TP FP TN FN		
2.	Pos Neg	Pos Neg	TP FP TN FN		
3.	Pos Neg	Pos Neg	TP FP TN FN		
4.	Pos Neg	Pos Neg	TP FP TN FN		
5.	Pos Neg	Pos Neg	TP FP TN FN		
6.	Pos Neg	Pos Neg	TP FP TN FN		
7.	Pos Neg	Pos Neg	TP FP TN FN		
8.	Pos Neg	Pos Neg	TP FP TN FN		
9.	Pos Neg	Pos Neg	TP FP TN FN		
10.	Pos Neg	Pos Neg	TP FP TN FN		

Total TP ____ Total FP ____ Total TN ____ Total FN ____

Total number of slides agreed (total TP + total TN) ____

Percent agreement (Total TP + Total TN) * 100 / Total number of slides reread

Monthly Report on Laboratory Confirmed Malaria Cases

Refer to Laboratory Register

Microscopy

Date (Month/Year)

Number of blood slides examined

Number of positive blood slides

Refer to Laboratory Register

RDTs

Date (Month/Year)

Number of RDTs tested

Number of positive RDT

Prescriber Adherence with Negative Malarial Tests

	Test type		Antimalarial prescribed		
**1.**	**RDT**	**Blood Slide**	**Yes**	**No**	**If prescribed, name of drug**
**2.**	**RDT**	**Blood Slide**	**Yes**	**No**	**If prescribed, name of drug**
**3.**	**RDT**	**Blood Slide**	**Yes**	**No**	**If prescribed, name of drug**
**4.**	**RDT**	**Blood Slide**	**Yes**	**No**	**If prescribed, name of drug**
**5.**	**RDT**	**Blood Slide**	**Yes**	**No**	**If prescribed, name of drug**
**6.**	**RDT**	**Blood Slide**	**Yes**	**No**	**If prescribed, name of drug**
**7.**	**RDT**	**Blood Slide**	**Yes**	**No**	**If prescribed, name of drug**
**8.**	**RDT**	**Blood Slide**	**Yes**	**No**	**If prescribed, name of drug**
**9.**	**RDT**	**Blood Slide**	**Yes**	**No**	**If prescribed, name of drug**
**10.**	**RDT**	**Blood Slide**	**Yes**	**No**	**If prescribed, name of drug**

Total number of negative RDTs

Total number of negative blood slides

Total number of patients with a negative malaria test and an antimalarial medicine prescribed

Total number of patients with a negative malaria test and NO antimalarial medicine prescribed | |
